# Three-dimensional Printer Molds for Vaginal Agenesis: An Individualized Approach as Conservative Treatment

**DOI:** 10.1055/s-0042-1756214

**Published:** 2022-09-22

**Authors:** Marina Silva Fernandes, Claudia Cristina Takano, Thyeres Teixeira Bueno Chrispin, Gisele Vissoci Marquini, Manoel João Batista Castello Girão, Marair Gracio Ferreira Sartori

**Affiliations:** 1 Escola Paulista de Medicina, Universidade Federal de São Paulo, São Paulo, SP, Brazil

**Keywords:** 3D printing, Mayer-Rokitansky-Küster-Hauser syndrome, vaginal agenesis, vaginal dilation, impressão 3D, síndrome de Mayer-Rokitansky-Küster-Hauser, agenesia vaginal, dilatação vaginal

## Abstract

**Objective** The aim of this study was to evaluate the use of vaginal molds, made with three-dimensional (3D) printing, for conservative treatment through vaginal dilation in patients with vaginal agenesis (VA).

**Methods** A total of 16 patients with a diagnosis of VA (Mayer-Rokitansky-Küster-Hauser syndrome, total androgen insensitivity syndrome, and cervicovaginal agenesis) from the Federal University of São Paulo were selected. Device production was performed in a 3D printer, and the polymeric filament of the lactic polyacid (PLA) was used as raw material. A personalized treatment was proposed and developed for each patient.

**Results** There were 14 patients who reached a final vaginal length of 6 cm or more. The initial total vaginal length (TVL) mean (SD) was 1.81(1.05) and the final TVL mean (SD) was 6.37 (0.94); the difference, analyzed as 95% confidence interval (95% CI) was 4.56 (5.27–3.84) and the effect size (95% CI) was 4.58 (2.88–6.28).

**Conclusion** The 3D printing molds for vaginal dilation were successful in 87.5% of the patients. They did not present any major adverse effects and offered an economical, accessible, and reproducible strategy for the treatment of VA.

## Resumo

**Objetivo** O objetivo deste estudo foi avaliar o uso de moldes dilatadores vaginais, confeccionados com impressão tridimensional (3D), para tratamento conservador através da dilatação vaginal em pacientes com agenesia vaginal (AV).

**Métodos** Foram selecionadas 16 pacientes com diagnóstico de AV (síndrome de Mayer-Rokitansky-Küster-Hauser, síndrome de insensibilidade androgênica total e agenesia cervicovaginal), da Universidade Federal de São Paulo. A produção dos dispositivos foi realizada em uma impressora 3D e, como matéria-prima, foi utilizado o filamento polimérico do poliácido lático (PLA). Um tratamento personalizado foi proposto e desenvolvido para cada paciente.

**Resultados** Quatorze pacientes atingiram um comprimento vaginal final (CVF) de 6 cm ou mais. A média inicial do CVF (DP) foi de 1,81 (1,05) e a média final do CVF (DP) 6,37 (0,94); a diferença (IC 95%) foi de 4,56 (5,27–3,84) e o tamanho do efeito (IC 95%) foi de 4,58 (2,88–6,28).

**Conclusão** Os moldes de impressão 3D para dilatação vaginal obtiveram sucesso em 87,5% das pacientes. Como impacto secundário, não apresentaram efeitos adversos importantes e ofereceram uma estratégia econômica, acessível e reprodutível para o tratamento da AV.

## Introduction

Vaginal agenesis (VA) is a congenital malformation and 90% of cases are associated with Mayer-Rokitansky-Küster-Hauser syndrome (MRKHS). The MRKHS has an incidence ranging from one case for every 4,000 to 5,000 female births, and is characterized by the congenital absence of the uterus and the upper ⅔ of the vagina. 1 Such patients have a female karyotype (46XX), function ovarian and normal sexual characters. Differential diagnosis should be made for patients with a transverse vaginal septum and an imperforate hymen. 1 2

According to the American College of Obstetricians and Gynecologists (ACOG), the treatment for VA can be conservative or surgical, and has the objective of restoring the vagina's anatomy and function. Vaginal dilation is the method used in the conservative treatment of VA. 3 4 5 This method was developed by Frank in 1938 and modified by Ingram in 1981, reaching an effectiveness of approximately 90%. 3 4 5 It is performed using progressively larger sized rigid vaginal molds until the proper vaginal length is achieved.

While there is no consensus on the best therapeutic strategy, the ACOG and most of the scientific community recommend that the least invasive and effective treatment should be adopted, with vaginal dilation being the first line of treatment for VA. 3 4 5 Additionally, conservative treatment through vaginal dilation is the first choice due to the good results and low rate of complications. 5

Three-dimensional (3D) printing is gaining wide use in the health care field. Especially in gynecology, it is possible to manufacture various devices, such as pessaries and vaginal molds, using a wide variety of materials. 6 Using this technology, devices that are fit for purpose and cost-effective are created. The objective of this study was to evaluate the use of personalized vaginal molds made with 3D printing for conservative treatment through vaginal dilation in patients with VA. 1

## Methods

The study was performed at the Federal University of São Paulo (UNIFESP), between June 2017 and October 2019, after approval by the Human Research Ethics Committee of the same institution, under the Certificate of Presentation of Ethical Appreciation number (CAAE): 91233917–9 and opinion number 2970405. The interventions were only made after approval, with the aim of offering an individualized and conservative treatment for each patient who voluntarily proposed to participate in the study. The patients who were willing to participate in the study signed an informed consent form.

The present study protocol was purely observational, which obviated the need for registration on clinical trial platforms. Additionally, it was not a randomized study or clinical trial, due to the rarity of the pathology, requiring patients to make a clear choice for conservative treatment before the beginning of the study.

The patients were selected from the Female Genital Malformations Sector at UNIFESP according to the following inclusion criteria: confirmed diagnosis of VA due to SMRKH, androgen insensitivity syndrome (AIS), or cervicovaginal agenesis, and desire to undergo conservative treatment.

The exclusion criterion was not wanting conservative treatment or not having free will or availability to participate in the research. All patients underwent evaluation by a multidisciplinary team: physician, physiotherapist, and psychologist. All patients were properly advised about the anatomy of the external genitalia before treatment.

The study's protocol had three phases: prototype development, patient selection, and mold application. Characteristics, dimensions, and initial parameters were defined through research of devices already available on the market and adjusted for each patient.

The initial geometric parameters were defined (cylindrical mold with the tip tapering progressively). For modeling the prototypes, the AutoCAD (Autodesk Inc., Mill Valley, California, USA) and FreeCAD 3D parametric modeling software were used based on the requirements defined above; computer aided design (CAD) system is the generic name for software used by engineering, geology, geography, health systems, and architecture and design to facilitate technical design and drawing. 6

**Fig. 1:**
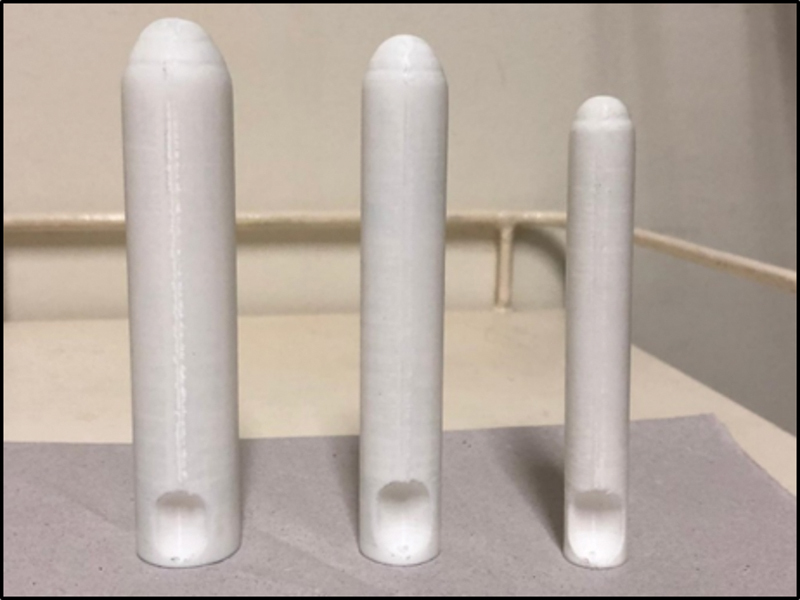
Vaginal dilators created with 3D printing. Source **:** Marta Maria Kemp.

The production of the devices was performed using the 3D cube printer, developed by the company 3D Systems (Rock Hill, SC, USA), using the polymeric filament of lactic polyacid (PLA) as raw material. The devices were evaluated by the medical staff for adjustments before application in the study. Three standard molds were created ( Fig. 1 ) with the following sizes (from right to left): Dilator A (1.5 × 8 cm); Dilator B (2 × 9 cm); and Dilator C (2.5 × 12 cm). 6

The individual molds were reused only by the patient herself. The molds were not sterile, but the patients were instructed to clean them with soap and water and use them with condom protection. During the test period, the authors realized that the sizes described above were the most used. These 3 sizes were used because there was no need to use larger or smaller sizes at any time. Despite the biological plausibility of having no contraindication for use in the supine position, as long as the patient is well oriented, the present study prioritized the systematic methodology of the same position of introduction of the casts in the gynecological position.

The applicability of the molds and the success of the vaginal dilation treatment were evaluated considering variables such as final total vaginal length (TVL), patient satisfaction, complication rate, and cost of mold production.

**Fig. 2:**
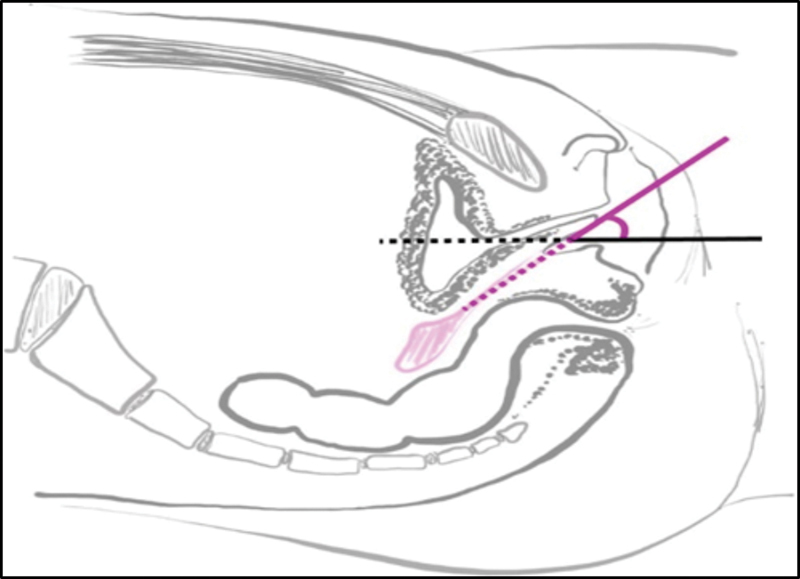
Positioning the dilator into vaginal introitus (purple). Source **:** Marta Maria Kemp.

The patients were instructed to perform light pressure exercises from the vaginal introitus, positioning as shown in Fig. 2 . The first return was within 15 days, and after that there was a monthly follow-up during the first year of the study. Patients who reached 6 cm or more in TVL were considered treated. After 6 cm of TVL, the patients were allowed to attempt sexual intercourse.

**Fig. 3:**
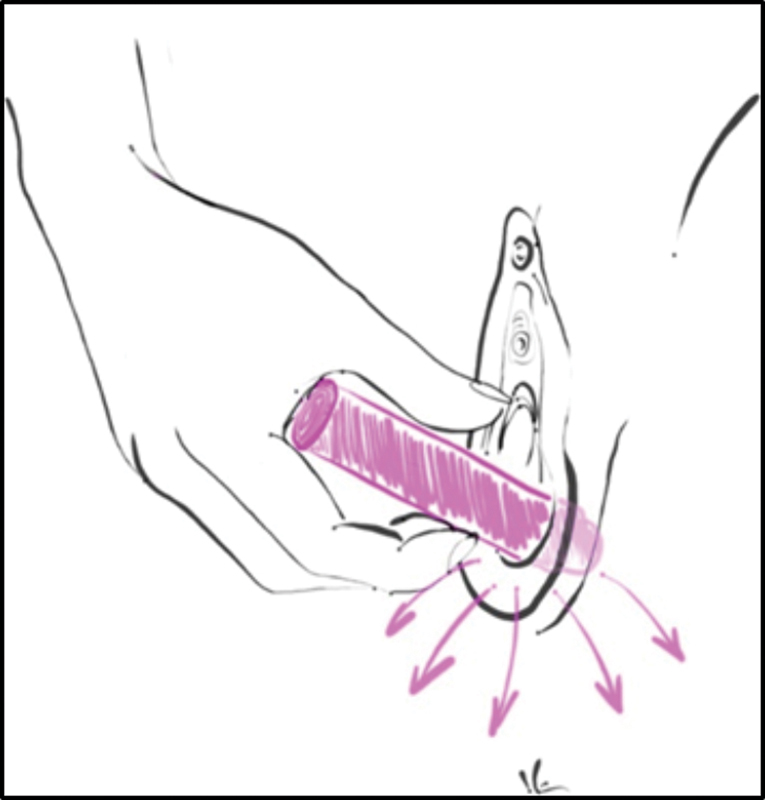
Orientation of perineal massage with the dilator. Source **:** Marta Maria Kemp.

The orientation of the direction and strength of the perineal massage vectors was standardized as introduction movements into the vagina and circular movements, toward the sides of the vagina and posterior wall or toward the perineal region, to preserve possible urethral trauma in the anterior region, according to Fig. 3 , for 20 minutes during the study. The strength guidance was individualized, being gradual and progressive according to the sensitivity to pain or discomfort of each patient.

All patients were followed up every month from the beginning of the treatment, during the 1 ^st^ year, and every 2 months in the 2 ^nd^ year of the study, with free return as needed. During follow-up, the newly created vaginal canal was analyzed using a speculum and by digital vaginal exam. The aspect, amplitude, and length were observed. All patients were allowed to have sexual intercourse when vaginal length was greater than 6 cm. The clinical aspects of the vagina were subjectively analyzed by the first two independent authors, with no difference regarding the appearance of the neovagina, such as presence or absence of active bleeding, the color of the vaginal mucosa, and granulation tissue in all patients, in the period of 3, 6, 12 and 24 months, and after treatment. The patients remain under follow-up at the same service with possible long-term results in future studies.

To compare the means of the initial and final TVL variable, the Student *t-* test for related samples was used. The D'Agostino normality test was performed for the assumptions of the analysis. To assess the magnitude of the difference, the effect size (d) with a 95% confidence interval (95% CI) was used. According to Cohen, 7 it was agreed that the values of d are considered small if (20 ≤ d < 50); medium if (50 ≤ d < 80); and large if (d ≥ 80).

## Results

A total of 16 patients were treated with the 3D printing dilators between October 2017 and October 2019. The patients in the present study had no exposure to previous treatments. The mean age was 19 years (standard deviation, SD: 2.84), mean initial vaginal length of 1.8 cm, and 60% of the patients had an associated malformation. Furthermore, 14 patients (87,5%) achieved a TVL greater than 6 cm at the end of the evaluation ( Table 1 ). The median time taken to reach treatment TVL was 5.6 months. The 2 patients who did not achieve TVL (patient number 15 and 16–Table 1) had used the molds for only 2 and 3 months, respectively.

**Table 1:** Initial and final TVL

Patient	Initial TVL (cm)	Final TVL (cm)
1	2	7
2	4	6.5
3	1.5	7
4	1	7
5	2	7
6	4	8
7	1.5	6
8	2	6
9	1	6
10	0.5	6.5
11	1	6
12	1	6
13	1.5	7
14	1	7
15	2	4
16	3	5

**Abbreviation:** TLV, total vaginal length.

As shown in Table 2 , there was a significant difference ( *p* < 0.05) between the initial and final TVL measurements. The effect size was 4.58, reinforcing the great magnitude of this difference.

**Table 2:** Statistical Analysis: Initial and Final TVL

Initial TVL mean (SD)	Final TVL mean (SD)	Difference (95% CI)	Effect size (95% CI)	*p* -value*
1.81 (1.05)	6.37 (0.94)	4.56 (5.27–3.84)	4.58 (2.88–6.28)	0.0001

**Abbreviations:** CI, confidence interval; SD, standard deviation; TLV, total vaginal length. **Note:** * *p* -values < 0.05 were considered statistically significant.

**Fig. 4:**
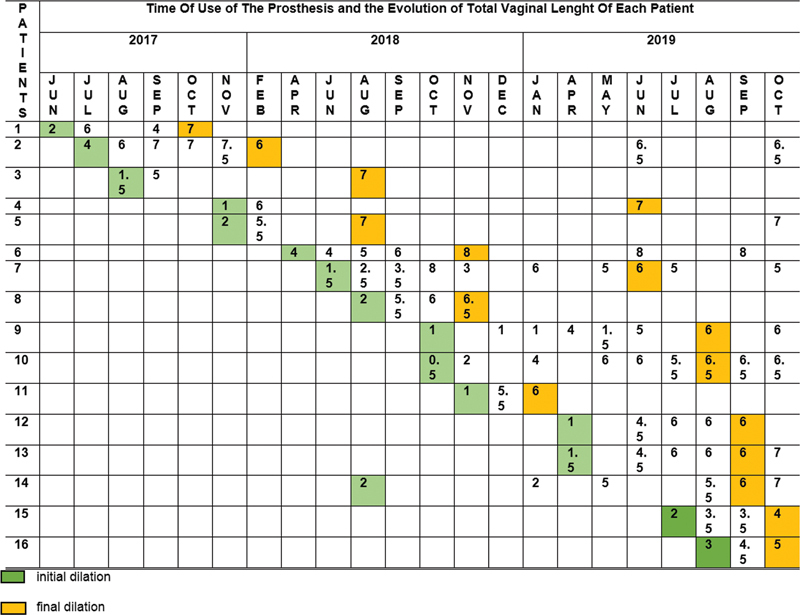
Time of use of the prosthesis and the evolution of total vaginal length of each patient.

The patients did not report any adverse effects, such as pain, discomfort, or bleeding, as they were instructed to perform dilation according to their ability, respecting their limitations of pain or discomfort. The authors believe that the absence of complications may be related to the previous educational guidelines and follow-up. Only 2 patients had inadvertent dilation of the urethra at the beginning of the treatment. Both had a smaller vaginal introitus (shorter distance between the urethral meatus and the vaginal furcula). They were reoriented in relation to anatomy and perineal massage exercises. After that, both were able to reach the treatment TVL. The patients were not operated on later because they were satisfied with the conservative treatment's results. They remain under follow-up at the specialized outpatient clinic of the same service for future follow-ups. Fig. 4 shows the evolution analysis of the time of prothesis use by each patient. According to these results, there was no statistically significant difference between the time of use of the 14 patients that patients who adhered and were successful during the evolution of the treatment ( *p* = 0.189).

## Discussion

In 1938, Frank 4 described the first conservative treatment for vaginal dilation using Pyrex (Corning Inc., Corning, NY, USA) tubes of gradually increasing sizes (0.8, 1.5, and 2.0 cm in diameter). This was used to force the mucous membrane into the vaginal introitus region. No incisions were required for this procedure. 4 The main criticism of this therapeutic modality is that it requires a special dedication from the patients, as the exercises with the dilators make it possible to create a vaginal canal that enables sexual intercourse. Maintaining a vaginal prosthesis is sometimes necessary to keep the vaginal canal patent, as well as performing exercises in the absence of regular sexual practice.

Decades after Frank's first description, several studies reported favorable results using his method. 8 9 10 11 In 1981, Ingram 12 suggested that the failures in the technique used by Frank were due to tiredness of the hands and fingers during the procedure, the need to use the embarrassing position, and the inability to perform other productive activities during the procedure. 10 In an attempt to overcome these limitations, Ingram 12 proposed a modification of the original Frank method. In the Ingram method, the patient's weight is used to replace manual and digital effort. The specially designed bicycle seat bench was used to facilitate perineal mold pressure. 12

Additionally, corroborating the results of the successful experiment by Ingram, 12 Roberts et al. 13 reported a 91% success rate using the Ingram method in their study of 51 patients with MRKHS. When well advised and emotionally prepared, almost all patients (90–96%) will achieve a satisfactory anatomical and functional result with vaginal dilation. 5 A recent study performed at the same reference center of the present project compared, in terms of anatomical, functional, and sexual aspects, two types of treatment for women with VA: progressive dilation (using the Frank method) or surgical neovaginoplasty (using the modified Abbé-McIndoe technique with oxidized cellulose). According to that study, both treatments had satisfactory efficacy and positive outcomes regarding the analyzed aspects. These data reinforce the reliability of the results from the present study, which indicate that dilation treatment can remain the first-line therapy for VA. 14

The literature lacks more consistent and robust studies comparing the different surgical techniques with each other and with vaginal dilation. However, so far, no surgical technique has surpassed the success rate of nonsurgical treatment; a fact that, together with the benefit of being a safer technique, places vaginal dilation as the first line in the treatment of VA. 5 15 16 17 18 19 20 21 22 In this context, the present study suggests the development of a personalized conservative treatment for each patient through vaginal dilation with 3D molds.

In 1984, Charles Hull 23 founded the world's first 3D printing company, with the use of production technologies such as Additive Manufacturing (AM) and Rapid Prototyping. The AM is used in the synthesis of a given physical object by adding layers to form a part based on data generated by CAD. These technologies are widely used to quickly prototype products and tools for commercial purposes. Over time, it has also been integrated into other areas, such as the health area, since these tools have enabled the assistance of health professionals in diagnosis, surgical planning, and synthesis of orthotics and prostheses for the rehabilitation of patients. 23

The use of 3D printing is gaining considerable acceptance in many medical fields, including surgery. The resulting tactile feedbacks significantly help the comprehension of anatomical details, especially the spatial relations between structures. Currently, an increasing number of applications have been successfully tested in many surgical disciplines, extending the range of possible uses to preoperative planning, counselling with patients, education of students and residents, surgical training, intraoperative navigation, and others. 23 24

In a recent systematic review, Barbosa et al. assess previous publications within 3D printing in human reproduction and gynecology. Based on the included studies, it was possible to design 3D models (uterus, ovaries, uterine cervix, and uterus with fibroids) that provided enriched information to improve presurgical planning, medical training, fertility-sparing surgery, patient comprehension of surgical procedures, and assisted reproduction applications. 25

Future expectations for 3D printing concern the reduction of manufacturing costs and time to further increase accessibility, as well as the development of novel techniques and suitable materials for biological structures, making it possible to recreate the architecture and functionality of real human organs and tissues. 23 24

The choice of devices made using a 3D printer was based on the possibility of offering an individualized treatment for each patient at a low cost and with a low rate of complications, in line with the plausibility of mold development already demonstrated in other areas, such as in Gynecology. The devices can be made with the most diverse formats and materials, which allows them to be adapted to the needs of each patient. Of the 16 patients treated, 14 reached the vaginal length considered for treatment, representing an 86% success rate. The only 2 patients who did not achieve a TVL of 6 cm or greater were still starting treatment (only 2 and 3 months ago).

According to the results of this present study, as well as in the literature, 5 15 16 17 18 19 20 21 22 conservative treatment for VA remains an excellent choice, with good efficacy and few complications, through personalized vaginal molds made with 3D printing. These results highlight the good applicability of the devices, bringing a cost-effective and easily reproducible option for the treatment of VA, making this a promising and accessible tool. Therefore, it would be a fruitful option to facilitate its use over the country, train professionals to apply the treatment, and shorten the distances so that more patients could benefit from it, thus eliminating the bias of distance and regularity in performing the exercises.

The main relevance of this research is the possibility of offering an individualized treatment option that is recommended in the scientific community with ethics, efficacy, and safety for a patient in an international reference center for the treatment of VA. Additionally, this is the first study to analyze reproductible 3D molds with conservative treatment and improve sexual function in women with VA. The homogeneous patient sample, standardized procedures, and prospective model are also strong points.

Another positive impact of this project was the effective response to the guidance of perineal massage exercises with the cast in patients with smaller vaginal introitus. This strategy can be used before the beginning of the dilation itself, aiming to reduce the chance of inadvertent dilation of the urethra.

The present study was not a randomized trial because of ethical issues. The main limitation of this trial was the small sample size. However, VA is a rare disease, with an incidence of 1:4.000 female births. The seriousness and scientific effort of this study are not diminished because of the difficulty to include more patients.

The main difficulties encountered in this study were the lack of motivation, lack of privacy in the patients' home, and distance from the city of origin to the hospital. Another obstacle was the attendance to outpatient follow-ups, since many patients lived in different cities and some even in other districts, as well as the regularity in the performance of the exercises, which depended on the personal motivation of each patient, home privacy, and emotional situation during treatment.

Considering the statements above, the authors believe that the study's strengths overcome its limitations. As VA is a rare disease that affects young women and involves the sensitive issues of sexuality and self-esteem, disclosure of well-structured trials can contribute to gaining knowledge so that an increasing number of women can benefit from the results of the studies.

## Conclusion

Based on the present findings, a 3D model device can be offered in a personalized and individualized way as the first-line conservative treatment for VA in nonspecialized health centers in developed and developing countries. Furthermore, the use of 3D printing for making the molds proved to be a promising, effective and reproductible strategy, especially to be applied in health care centers with limited financial resources or a shortage of professionals specialized in the surgical treatment of VA, with low rate of complications. Considering the encouraging outcomes of this project and the rarity of the evaluated clinical condition affecting young women, the authors suggest more well-structured trials should be performed to better treat and benefit this population.
